# Thirty Years of the DICOM Standard

**DOI:** 10.3390/tomography9050145

**Published:** 2023-10-06

**Authors:** Michele Larobina

**Affiliations:** Istituto di Biostrutture e Bioimmagini, Consiglio Nazionale delle Ricerche (CNR), I-80145 Napoli, Italy; michele.larobina@cnr.it; Tel.: +39-081-2203410

**Keywords:** DICOM, metadata, file formats, communication protocols, quantitative imaging

## Abstract

Digital Imaging and Communications in Medicine (DICOM) is an international standard that defines a format for storing medical images and a protocol to enable and facilitate data communication among medical imaging systems. The DICOM standard has been instrumental in transforming the medical imaging world over the last three decades. Its adoption has been a significant experience for manufacturers, healthcare users, and research scientists. In this review, thirty years after introducing the standard, we discuss the innovation, advantages, and limitations of adopting the DICOM and its possible future directions.

## 1. Introduction

Standardization is a key concept in the digital imaging world. The absence of a standard limits the usability and sharing of the images. It forces users to deal with a multitude of data formats and to convert data from one format to another. Moreover, any image file, in addition to pixel data, contains metadata. Metadata is data describing the image and plays a non-secondary role in digital imaging. While general-purpose image format metadata can be limited to the description of the pixel matrix, in formats for scientific applications metadata can describe the subject, the instrumentation set-up, the image acquisition parameters, and any other element of interest related to the imaging workflow. Despite this, the power of metadata is most often underestimated and consequently unexpressed. A standard helps in defining the metadata section for the correct use and interpretation of the image itself.

The field of medical imaging is exemplary in the context of standardization processes for pioneering vision and for having created a long-lived and appreciated standard. In the early 1980s, an association of users and professionals of the healthcare sector, the American College of Radiology (ACR), jointly with the National Association of Electronic Manufacturers (NEMA), started to define a new standard for the encoding and exchange of digital medical images. In 1993 the ACR-NEMA committee presented the Digital Imaging and Communications in Medicine (DICOM) as a standard with more functionality and long-term vision than the previous standardization attempts known as ACR-NEMA 1.0 (1985) and 2.0 (1988) [1,2,3,4,5,6,7]. For its time, DICOM represented an authentic novelty. Before the introduction of the DICOM standard, and therefore, until the first half of the 1990s, the medical imaging world saw diagnostic modalities, even within the same department, very confined to their rooms. The images were generally printed to film to be interpreted by the radiologist. In a native digital format, images were viewed and processed on the modality console and rarely exported to different workstations. Medical imaging systems were not connected to each other except for some dedicated point-to-point connections. Image transfer between modalities and image processing workstation, both inside and outside an imaging department, took place mainly through removable media with an important limitation: the absence of a common file format and the unknown of correctly reading the removable media storage (typically a magneto-optical disks or a tape). Therefore, the usability of the images remained linked to the availability of software for reading proprietary image formats and the media themselves. A complete overview of the old proprietary file formats can be found on the David Clunie Medical Image Formats website [8]. The DICOM international standard was conceived to overcome the limitations imposed by proprietary architectures and data formats and to allow and promote communication among medical imaging devices using the same network infrastructures of the Internet computer networks. The novelties of the DICOM standard are:

- To state that, in medicine, the standardization of image format and image-related information, as well as their communication over a network, is essential.
- To remark that, for medical images, metadata is as important as pixel data.
- To be general enough to cover almost every medical imaging modality and flexible enough to follow their evolution over time.

First developed for radiology and then cardiology departments, the DICOM standard has evolved over the years to support various other branches of medical imaging well beyond radiology, such as dermatology and ophthalmology, with the objective to encompass almost all modalities of imaging-based medicine. There are approximately 80 modalities defined by the standard today. DICOM is also the current standard for radiation therapy in the so-called second-generation radiotherapy after a complete revision in 2014. DICOM also supports data exchange of time-based signals or waveforms, such as those generated in clinical neurophysiology, which include, among others, electrocardiograms (ECGs) and electroencephalography (EEG). In more recent times, the DICOM standard has been proposed for digital pathology. The adoption of the standard in this area would favor the integration of clinical imaging and laboratory medicine [9,10,11]. DICOM does not limit its action to images and associated information originating directly from medical devices. It defines mechanisms for archiving and sharing quantitative derived images, image-derived data, annotations, and reports.

Although DICOM only provides recommendations and no accompanying software, the availability of some high-quality open-source software libraries and utilities in several programming languages, such as DCMTK (C and C++), DCM4CHE (Java), and PyDICOM (Python) [12,13,14], has helped in spreading and affirming the standard.

DICOM is a constantly evolving standard and is revised five times a year with contributions from the numerous DICOM working groups divided by fields of application and imaging modalities [15].

This article presents an overview of the DICOM standard, covering its fundamental principles and concepts. It also explores the advantages and limitations of the standard while outlining some potential future developments. Throughout this document, all references to the DICOM standard in sections, figures, and tables refer to the 2023b edition.

## 2. Not Only Pixels: The Power of Metadata in Medical Images

Medical images must be standardized in a format that can be stored, shared, and used effectively. Therefore, a standard must necessarily deal with metadata as well. The DICOM standard aims to establish a reliable format for medical images and associated information. One of the most significant advancements of the DICOM image format is its metadata formulation, which provides an accurate and detailed description of the subject and procedure used to generate the image. The standard emphasizes that metadata is essential for the full use of medical investigation for clinical, management, and research purposes, establishing the non-divisibility of the pixel data from the metadata. Each DICOM image consists of metadata and pixel data embedded in a single file so that, as the standard institutional website remarks, “the image can never be separated from this information by mistake”.

**Figure 1:**
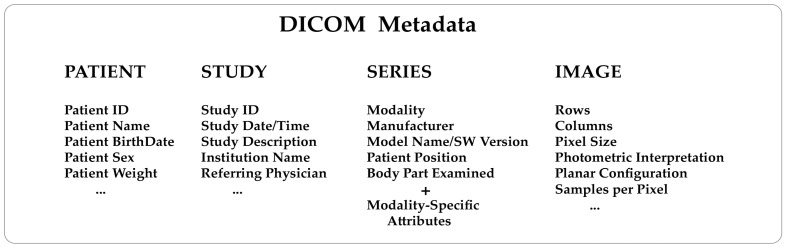
DICOM image metadata contains detailed information to identify and describe the main entities of the imaging workflow: Patient, Study, Series, and Images. The *Patient* data section essentially includes a unique patient identifier, the patient’s name, date of birth, sex, and data such as the patient’s weight, which is required to normalize voxel values by body weight as in the case of standardized uptake value (SUV) in PET. The *Study* section includes a unique identifier, the study date and time, the study description, the Institution name, the referring physicians, etc. The *Series* section will contain a unique identifier, the body part examined, the field of view, and data related to the imaging modality, such as acquisition protocol and scanning parameters, as well as the manufacturer name, the model, and the software of the equipment used. Finally, the *Image* section will contain a description of the pixel data necessary for the correct loading and display of the image: rows, columns, samples per pixel, bit depth, photometric interpretation, pixel size, etc.

Knowing how a medical image has been generated from the diagnostic modalities is extremely helpful. For example, a nuclear medicine image will contain information about the injected radiopharmaceutical, injection time, acquisition start time, and end time. An X-ray computed tomography image will contain information about the X-ray tube voltage and current, exposure time, slice thickness, etc. This kind of data are consistently included in the metadata of every DICOM image. To understand the structure of a DICOM metadata section, remember that the standard describes real-world entities, such as patients, studies, diagnostic modalities, and images, in terms of objects and relationships that may occur between them (the so-called entity-relationship model). It is a high level of abstraction that has helped make the DICOM a complex format. Objects are defined in a standard way through groups of attributes describing them in detail. As a result, DICOM needs to store many attributes (Figure 1) to guarantee a comprehensive depiction of the imaging process.

How is the metadata section populated? There are attributes set during the installation/configuration of the imaging modality (mainly related to the hardware and software of the system, the institution name, etc.), attributes automatically exchanged with the Hospital Information System (HIS)/Radiology Information System (RIS) of the department, attributes specified by the acquisition procedure selected for the examination, etc.

In a DICOM file, each attribute is identified by a unique tag consisting of two hexadecimal numbers. The first represents the group, and the second represents the element. For example, the tag for the modality is (0008, 0060).

DICOM attributes are logically grouped into modules to identify and describe real-world objects. Modules are presented in standard documents in the form of tables. Modules store the name, tag, definition, and type of the attributes and can hold attributes from various groups. There are modules common to multiple imaging modalities and others specific to one modality, as outlined in Annex C.7 and C.8 of the PS 3.3 standard document (Table 1). Groups of attributes repeated across multiple modules are called macros. The DICOM data dictionary (PS 3.6) contains all the attributes defined and described in the standard, known as public data elements. Metadata can also include manufacturer-specific attributes known as private data elements for which there is no description and are not part of the data dictionary. It is important to note that private data elements are assigned an odd group number, whereas public data elements always have an even group number. The number of data elements present in a DICOM file, both public and private, is generally variable. A specific tag indicates the start of the pixel data and hence the end of the metadata section.

Regarding the DICOM pixel data section, the support for floating point values (single precision 32-bit and double precision 64-bit) is limited to radiation dose values in radiotherapy and, more recently, to parametric maps defined as images in which the pixel values have been derived from the value stored by the modality to be the expression of a physical quantity. In all the other cases, DICOM pixel values can only be integers. DICOM uses a scale factor whenever the values stored in each voxel need to be scaled to different units. This is achieved through two fields specified in the metadata defining the slope and the intercept of the linear transformation to be used to convert pixel values to real-world values.

## 3. Rules and Tools for the Exchange of Medical Images and Related Information

Beyond a standardized file format for medical images and associated information, the DICOM standard provides a communication protocol to easily share images in a vendor-independent manner. Protocols are defined by Tim Berners-Lee, one of the creators of the World Wide Web, as *simple rules for global systems* [16]. This definition is both brief and impactful.

The purpose of the DICOM protocol is to establish communication between diagnostic and sometimes therapy systems of different manufacturers and display, storage, and management devices on a network. The introduction of the DICOM standard marks the beginning of a revolution similar to the introduction of computer networks: no more separate diagnostic equipment, but diagnostic systems, and in some cases of therapy, processing/display and reporting stations that can be connected together and that can share images and related data, storage devices and printers. To realize this objective, the DICOM standard provides an upper-layer protocol that runs over the well-established Internet standard protocol TCP/IP. Any DICOM-compliant device attached to the network is an Application Entity identified, in addition to the TCP/IP parameters (IP address, subnet mask, and port number) by a 16-character identification code called “Application Entity Title”. Application Entities can exchange services among themselves according to the Client/Server model that the DICOM renames Service/Provider; the requestor of a service is called Service Class User, while the provider is called Service Class Provider. Depending on the service, a DICOM node can act as a user or provider. Establishing an association involves a negotiation phase during which the service to be exchanged and the role played by each node is established. Next, the transfer syntax for the data exchange is decided, the connection is established and the data transfer takes place. The main services available on a DICOM network are listed in Table 2.

Recently, DICOM has added a protocol called DICOMweb built on top of the HyperText Transfer Protocol (HTTP) for using services via the web [17,18,19,20]. DICOMweb enables query, retrieval, storage, and worklist services. DICOM images can be retrieved traditionally as binary objects containing metadata and pixel data or with metadata in JavaScript Object Notation (JSON) or eXtensible Markup Language (XML) and pixel data as DICOM bulk data or, optionally, in a format suitable to be directly displayed in a web browser (JPEG).

**Figure 2:**
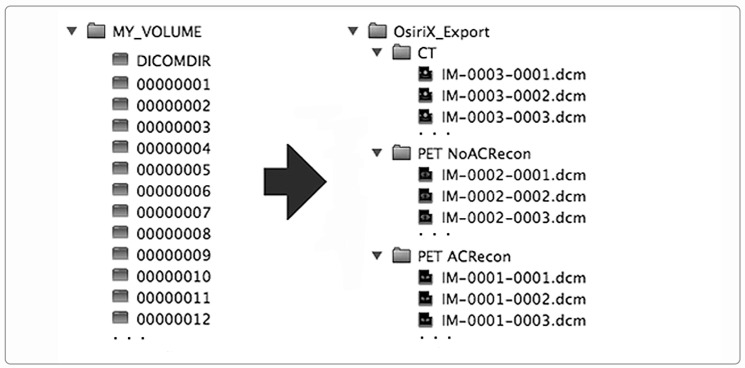
On the left, the DICOMDIR flat image files organization in the case of a patient who underwent a whole-body PET/CT diagnostic study. On the right, the corresponding image file organization using the hierarchical folder tree option (patient-study-series) in the OsiriX Viewer export utility. The PET study was reconstructed with and without Attenuation Correction (AC). According to the standard, DICOMDIR image filenames are no more than eight characters without any extension.

In addition to the storage of images through a network, the DICOM standard specifies how to standardize image storage on removable physical media such as CD/DVD/Blue Ray Disks, Magneto-Optical Disks, USB-Connected Removable Devices, and Compact-Flash Removable Devices (DICOM PS 3.12). In this way, the interoperability is extended and ensured even when the image exchange takes place through removable supports. To store DICOM images on physical supports, the standard prescribes a flat-file organization consisting of a folder that serves as a container for the patient’s images, along with a DICOMDIR file. DICOMDIR contains the association between image files and the patient study-series information for all the DICOM files on the media. DICOMDIR is not human-readable as it is a binary file in DICOM format, so a software utility is necessary to read it. Figure 2 shows the DICOMDIR concept schematically.

## 4. DICOM’s Strengths and Weakness

The DICOM is a complex standard designed to offer maximum flexibility with the ambition to virtually embrace almost all medical imaging modalities. The adoption of the DICOM standard has greatly improved the access, exchange, and usability of medical images. Today, PACS, RIS, and HIS are critical components of every imaging department, and their existence relies on DICOM. The power of metadata in medical imaging has been for a long time underestimated and consequently unexpressed. Thanks to DICOM, clinicians and researchers recognized, after an initial slow acknowledgment phase, that metadata are not only helpful but essential for a better understanding and management of the images. DICOM confirmed the assumption that metadata is as important as pixel data. The adoption of DICOM also encouraged and facilitated data exchange between researchers, creating added value for research. The DICOM standard also has a primary role in the emerging field of Enterprise Imaging, whose ultimate goal is to connect as many technologies as possible in a collaborative workflow in order to provide added value for the electronic health record [21,22].

### 4.1. Conformance (the DICOM Philosophy)

It is essential to understand that compliance with the DICOM standard is voluntary. The standard publishes the recommendations leaving the manufacturers free to implement or not some aspect of the standard, with the only duty to declare it in a Conformance Statement document to be left to the user. There is no certification or validation mechanism to verify compliance. This extreme flexibility ended up creating problems due to a consequent variability between the implementations of the various vendors, which led to a decrement in the level of interoperability between imaging systems and between imaging systems and PACS, especially in the earlier implementation of the standard.

Standards prescribe rules, but it is important to acknowledge that not all vendors, programmers, and researchers may consistently follow all suggested rules. Additionally, errors may occur due to the intricate nature of the standard.

It is clear that having a software tool to easily verify compliance with a standard is a helpful way to support and speed up the assertion of the standard itself. Such a test and validation tool becomes even more necessary as the complexity of the standard increases.

### 4.2. Private Tags

The presence in the metadata of private tags identifying private data elements is another peculiar aspect of the standard. As reported in Clunie et al. [23] “private data are data elements that the DICOM standard allows to be included, but whose meaning and encoding are not defined by the standard itself”. It is not difficult to understand that the use of proprietary data can create troubles besides appearing contradictory for an open standard. The DICOM standard considers private data necessary because manufacturers may need to codify and include in the imaging process description parameters or information not yet contemplated by the standard (PS 3.5—Section 7.8), as can be, for example, those related to innovative technological solutions adopted by instruments they produce. It is, therefore, a guarantee of maximum flexibility that the standard offers to the manufacturers and belongs to the long-term vision of the standard. This is the theory because, in practice, there are manufacturers who make excessive use of private tags, using them despite the existence of public tags intended to hold the same information [24,25,26,27]. Indeed, the use of private data could be better regulated and documented. Their overuse could have been classified as violating the standard without harming the trade-secret protection or affecting the generality of the standard. Unfortunately, the DICOM standard has not foreseen limitations for private tags, leaving the matter to be regulated based on the relationship between customers and device vendors.

### 4.3. Data Protection (Privacy)

Another issue connected to the adoption of the DICOM standard is that a metadata section rich in information with explicit reference to patients’ personal data poses a privacy problem every time the images are viewed or exported outside of the structure where they were acquired and reported. It is recommended to anonymize DICOM images before transmitting them externally. However, it is common experience, especially in the post-processing field, that sometimes anonymized images do not work while native ones do. This may be because certain processing software uses private information for measurements and calculations. Tools for de-identification/anonymization are numerous, but it is always necessary to carefully select the options that the de-identification software provides, limiting the fields that are overwritten (cleaned) and retaining whenever possible private tags even in the anonymized version as they could contain essential information for image analysis. The DICOM standard recently published a list of “known to be safe” private tags (PS 3.15 Annex E), which is a good practice to keep in the de-identification process [23].

Nevertheless, the goal of de-identification is not straightforward due to the need to satisfy the privacy regulations on the one hand and the capability to have a fully functional anonymized DICOM on the other. These difficulties have complicated data sharing between working and research groups and limited the creation of public databases containing DICOM images [28,29].

### 4.4. Quantitative Image Analysis

The primary focus of the DICOM standard is the clinical domain. The DICOM file format was not designed with image post-processing as an application. DICOM as image format is used by vendor-supplied quantitative processing tools, by groups that have developed their own software and are familiar with the standard, and whenever the processing relies entirely on a single software application that is compatible with DICOM. In other cases, researchers and programmers prefer to work with alternative formats, starting the post-processing pipeline by converting DICOM images into the format of choice for their research project [30]; then, they do not consider the DICOM format for encoding their analysis results, though, in recent years, software tools have been made available to the research community to facilitate the translation of the results of their analysis in the DICOM format [31,32]. DICOM remains the reference for the information reported in his metadata section. Cases in which metadata does not completely match researchers’ expectations are still encountered. Critical issues have been highlighted in magnetic resonance imaging, particularly for perfusion and diffusion studies. Commonly reported issues about metadata are, among others, an insufficient detail of information, not mandatory public attributes of interest, and the use of private tags to contain parameters essential for quantitation [24,26,33]. The lack of response to these needs has encouraged the development of alternative standards, such as the brain imaging data structure (BIDs) [34], that may not have been born if DICOM had shown greater openness towards the research world.

Computerized image analyses and processing may have even more relevance in the coming years due to the contribution that Artificial Intelligence techniques are expected to have. From a predominantly research activity separated from the clinical domain, image processing will be in the future increasingly integrated into clinical practice.

The DICOM standard has already developed tools to support quantitative imaging. Among these, Parametric Maps for storing images quantitatively derived from acquired images, DICOM Segmentation for the saving of segmentation results in terms of images, Structured Reports and Annotations for the encoding of image-derived associated data as volumes of segmented regions, measurements for cancer lesions characterization, etc. These DICOM tools still have limited support and adoption and might form the basis for future expansions of the standard. Undoubtedly, the support for image processing and the integration of image analysis results into PACS systems or enterprise image archives is a major challenge for the DICOM standard in the next decade.

## 5. Conclusions and Future Directions

DICOM demonstrates the positive influence and added value that a standard can have in a specific field. There is no standard for all areas of scientific imaging. For example, there is no single standard for biological image data despite numerous attempts to develop and adopt a standard. One of the first microscopy data formats, the Image Cytometry Standard (ICS), released in 1990 [35], was inspired by the work of the ACR-NEMA consortium and shows similarity to the Interfile format developed in the same years for nuclear medicine diagnostic images [36]. More recently, the Open Microscopy Environment Consortium (OME) first proposed a modified version of the well-known Tagged Image File Format (TIFF) with an enriched metadata section encoded in XML (OME-TIFF) [37], then, in an attempt to have a more complete and powerful format, has proposed his successor, the OME-Zarr [38]. The DICOM standard has also focused on the histopathology whole slide imaging application. It was chosen as the format for the Imaging Data Common project, a cloud-based imaging data science platform for cancer studies started by the US National Cancer Institute [39]. Other formats have been developed and successfully established in specific fields, such as the MRC format for cryo-electron microscopy [40] and the imzML for mass spectroscopy images [41]. Unfortunately, none of the proposed standards have been affirmed except for some specific fields, and vendor proprietary formats are still in use.

The possibilities for the affirmation of a standard lie in the convergence between industry and the scientific community. Sometimes, standards have been proposed by the scientific community but then have failed to establish themselves, probably because the industries of the sector have not recognized the advantages of a single standard to justify an investment or a change in the company policy on the subject.

The field of medical imaging is exemplary in the standardization processes for the pioneering vision and longevity of its standard promoted at the end of 1980. This experience highlights that the opportunity to have a standard should not be underestimated. Beyond revolutionizing the clinical practice, the DICOM standard has encouraged and facilitated data exchange between researchers, creating added value for research. We think adopting a standard in other imaging-based contexts could also have a similar positive impact.

Today, it is impossible to imagine an imaging department without DICOM. However, after 30 years, the time is ripe to review some of the initial directives. A modern standard should anticipate innovation, not just follow it. Overall, the DICOM should recover the initial long-term vision that allowed it to propose solutions ahead of time. Through experience, it has become clear that there are areas in which the DICOM standard could be further improved, including simplifying and clarifying the documentation, limiting and regulating private tags, increasing the support for research and quantitative image processing, and strengthening privacy protection procedures. Additionally, the development and use of validation tools should be encouraged to minimize non-standard implementations.

## Figures and Tables

**Table 1:** Summary of the modules defined in the DICOM standard document PS 3.3—Annex C7 and C8. The list of modality-specific modules has been truncated for brevity; there are approximately 80 modalities defined by the standard, each with its own separate module.

C.7. Common Composite Image IOD Modules	C.8. Modality-Specific Modules
C.7.1. Common Patient IE Modules	C.8.1. Computed Radiography Modules
C.7.2. Common Study IE Modules	C.8.2. CT Modules
C.7.3. Common Series IE Modules	C.8.3. MR Modules
C.7.4. Common Frame of Reference IE Modules	C.8.4. Nuclear Medicine Modules
C.7.5. Common Equipment IE Modules	C.8.5. Ultrasound Modules
C.7.6. Common Image IE Modules	C.8.6. Secondary Capture Modules
C.7.7. (Retired) Patient Summary Module	C.8.7. X-ray Modules
C.7.8. (Retired) Study Content Module	C.8.8. Radiotherapy Modules
C.7.9. Palette Color Lookup Table Module	C.8.9. PET Modules
C.7.10. Common Acquisition IE Modules	…
C.7.11. Common Multi-Resolution Pyramid IE Modules	C.8.32. Parametric Map
	C.8.33. Tractography Results Modules

**Table 2:** Main services available on a DICOM network.

*Storage*: it is the service required to archive images across a network. Typically, it is used by an acquisition modality to send images to a picture archiving and communication system (PACS) or a storage server.
*Storage Commitment*: it is an enhanced version of the Storage service with in addition a message sent by the storage provider to the user to confirm that “archiving was successful”, so that the user can safely delete the images locally.
*Print*: it is the service for printing images from an acquisition modality or a display station.
*Query/Retrieve*: it is the service enabling nodes on the DICOM network to query a picture archiving and communication system (PACS) or another storage unit in order to know the list of images available on it and then to retrieve them.
*Modality worklist*: it is the service able to manage the list of exams to be acquired for each patient. Each examination of the list is scheduled and its completion is made known to the system that updates the data. It is only possible in departments equipped with a computerized reservation/acceptance system (HIS/RIS) integrated into the DICOM network.

## Data Availability

Data sharing not applicable.

## Abbreviations

ACR	American College of Radiology
NEMA	National Electrical Manufacturers Association
OME	Open Microscopy Environment
DICOM	Digital Imaging and Communications in Medicine
TIFF	Tagged Image File Format
JPEG	Joint Photographic Experts Group
ICS	Image Cytometry Standard
BIDs	Brain Imaging Data Structure
PACS	Picture Archiving and Communication Systems
HIS	Hospital Information Systems
RIS	Radiology Information Systems
TCP/IP	Transmission Control Protocol/Internet Protocol
HTTP	HyperText Transfer Protocol
IOD	Information Object Definition
IE	Information Entity
MRI	Magnetic Resonance Imaging
CT	Computed Tomography
PET	Positron Emission Tomography
SUV	Standardized Uptake Ratio
JSON	JavaScript Object Notation
XML	eXtensible Markup Language.
